# *CDK4* Loss of Function Mutations Cause Microcephaly and Short Stature

**DOI:** 10.1101/gad.352311.124

**Published:** 2025-04-10

**Authors:** Aitana Verdu Schlie, Andrea Leitch, Maria Izabel Arismendi, Colin Stok, Andrea Castro Leal, David A. Parry, Antonio Marcondes Lerario, Margaret E. Harley, Bruna Lucheze, Paula L. Carroll, Kamila Musialik, Julia M. T. Auer, Carol-Anne Martin, Lukas Gerasimavicius, Alan J. Quigley, Joya Emilie de Menezes Correia-Deur, Joseph A. Marsh, Martin A. M. Reijns, Anne K. Lampe, Andrew P. Jackson, Alexander A. L. Jorge, Lukas Tamayo-Orrego

**Affiliations:** 1 MRC Human Genetics Unit, Institute of Genetics and Cancer, University of Edinburgh, Edinburgh, EH4 2XU, UK; 2 Genetic Endocrinology Unit (LIM25), Endocrinology Division, Faculdade de Medicina da Universidade de São Paulo (HC-FMUSP), São Paulo, Brazil; 3 Department of Integrated Health, State University of Para, Santarem, Brazil; 4 Division of Metabolism, Endocrinology, and Diabetes, Department of Internal Medicine, University of Michigan, Ann Arbor, MI, USA; 5 Paediatric Imaging Department, Royal Hospital for Children and Young People, Edinburgh, EH16 4TJ, UK; 6 South East of Scotland Clinical Genetics Service, Western General Hospital, Edinburgh, EH4 2XU, UK

## Abstract

Cell number is a major determinant of organism size in mammals. In humans, gene mutations in cell cycle components result in restricted growth through reduced cell numbers. Here we identified biallelic mutations in *CDK4* as a cause of microcephaly and short stature. *CDK4* encodes a key cell cycle kinase that associates with D-type cyclins during G1 of the cell cycle to promote S-phase entry and cell proliferation through retinoblastoma (RB) phosphorylation. CDK4 and CDK6 are believed to be functionally redundant and are targeted jointly by chemotherapeutic CDK4/6 inhibitors. Using molecular and cell biology approaches, we show that functional CDK4 protein is not detectable in cells with *CDK4* mutations. Cells display impaired RB phosphorylation in G1 leading to G1/S-phase transition defects and reduced cell proliferation, consistent with complete loss of cellular CDK4 enzymatic activity. Together, these findings demonstrate that CDK4 is itself required for cell proliferation, human growth and brain size determination during development.

## Introduction

Primary microcephaly is a Mendelian form of microcephaly where brain growth is markedly reduced during development and occurs in the absence of major malformations or neurological deficits (Verloes et al. 1993; Farcy et al. 2023). Mutations in multiple genes involved in centrosome and mitotic spindle biology were initially identified in primary microcephaly, leading to the proposal that defective neural stem cell mitosis causes microcephaly (Thornton and Woods 2009). However, other processes such as cell signalling, transcription, and the DNA damage response are also compromised in primary microcephaly (Faheem et al. 2015; Jayaraman et al. 2018), suggesting additional mechanisms of brain growth regulation.

Microcephalic dwarfism represents a group of genetic growth defects where both brain and stature are compromised, and is caused by mutations disrupting cell proliferation, genome stability, DNA replication and mitosis (Klingseisen and Jackson 2011). Multiple primary microcephaly (PM) genes have subsequently been linked to microcephalic dwarfism (MD) phenotypes, indicating that these two conditions should be considered as a phenotypic continuum (Shaheen et al. 2019; Asif et al. 2023).

Alterations in cell cycle genes are major drivers of human cancer (Malumbres and Barbacid 2009). However, despite the availability of multiple mouse models, cyclins and CDKs have not been extensively studied in the context of human growth. CDK4 and CDK6 are functionally redundant regulators of G1 progression and the G1/S transition (Matsushime et al. 1994; Kato 1999; Sherr and Roberts 2004). CDK4 and CDK6 form complexes with D-type cyclins and link extracellular growth signals with cell cycle progression and growth (Malumbres and Barbacid 2009; Romero-Pozuelo et al. 2020; Fassl et al. 2022). CDK4/6 promotes phosphorylation and inactivation of the RB tumour suppressor (Lundberg and Weinberg 1998; Harbour et al. 1999; Narasimha et al. 2014), resulting in E2F-driven transcription, increased CDK2/Cyclin E activity, and G1/S progression (DeGregori et al. 1995). Unsurprisingly given their anti-proliferative properties, CDK4/6 inhibitors are used as therapies against multiple cancers (Fassl et al. 2022; Morrison et al. 2024).

Here we report the identification and functional characterisation of biallelic loss of function (LOF) mutations in *CDK4* in individuals with microcephalic dwarfism. *CDK4* variants result in aberrantly spliced transcripts and undetectable full-length protein in patient fibroblasts, leading to impaired retinoblastoma phosphorylation in G1, delayed G1/S transition, and reduced proliferation, to explain the growth deficiency observed in the affected individuals.

## Results

### Identification of biallelic variants in *CDK4*

**Figure 1:**
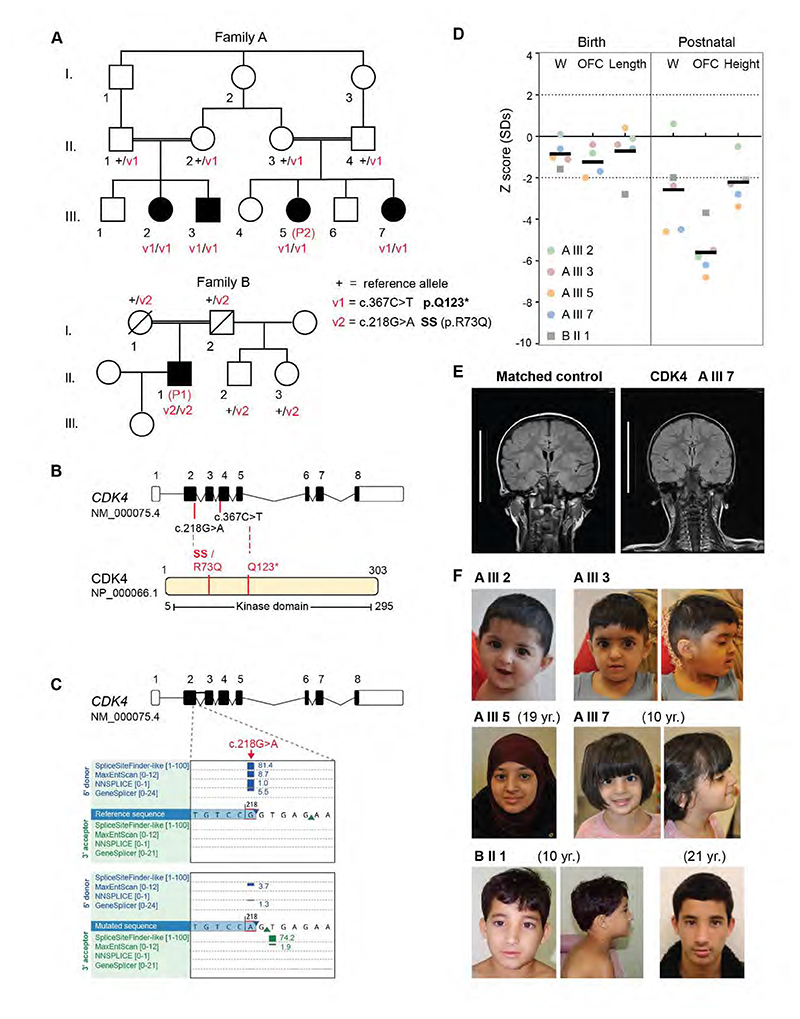
Individuals with biallelic *CDK4* variants display microcephaly and short stature. (*A*) Family pedigrees with segregation of *CDK4* variants; (Square) male, (circle) female, (filled symbols) individuals with microcephaly, (strike through) deceased. WT Reference (+), variants v1 and v2, and zygosity indicated for each studied individual. (*B*) Diagram of *CDK4* transcript (top) and protein; coding exons are depicted as black rectangles. Red lines indicate variant location; SS, splice site disrupted. (*C*) Altered splicing predictions for the c.218G>A substitution generated using Alamut. Blue rectangles indicate strength of splice donor predictions for individual splice algorithms, blue triangle, predicted donor splice site. (*D*) Growth parameters at birth and at last assessment (Postnatal); W, weight; OFC, orbito-frontal circumference. Z scores, standard deviations from population mean for age and sex. Dashed lines indicate 95% confidence interval for general population. Individual subject data points from Family A (circles) and B (square) are graphed, mean values plotted. (*E*) MRI scan of age-matched control (4 years and 8 months) and affected individual with *CDK4* variant; coronal FLAIR projection shows simplified parietal and temporal gyri, reduced white matter volume and absence of brain malformations. Scale bar, 10 cm; see also Supplemental Fig. S1C for additional MRI projections. (*F*) Photographs of all affected individuals.

We ascertained a consanguineous family of British Pakistani origin with four affected individuals presenting with extreme microcephaly and (three out of four) significant short stature (Fig. 1A, Tables 1,2). Whole genome sequencing of parents and affected individuals revealed the homozygous substitution c.367C>T in cyclin-dependent kinase 4 (*CDK4*; NM_000075.4) as the likely cause of the phenotype. This variant introduces a premature stop codon (p.Q123*) within the kinase domain of the protein (Fig. 1B), with its position in exon 4 of 8 predicting it also to result in nonsense-mediated decay of the transcript.

Through GeneMatcher (Sobreira et al. 2015), we then identified an individual of Brazilian origin with history of prenatal-onset growth restriction who also presented with marked microcephaly and short stature (Fig. 1A,B, Tables 1,2). Independent whole exome sequencing identified a homozygous c.218G>A variant in *CDK4*. This results in an arginine to glutamine substitution at codon 73 (p.R73Q), a conserved residue in mammals and most vertebrates (Supplemental Fig. S1A). Significantly, this nucleotide substitution also occurs at an essential splice site sequence, the last base pair of exon 2. Analysis of this variant using SpliceAI (Jaganathan et al. 2019), MaxEntScan and NNSPLICE predicts loss of the splice donor site located 1bp downstream of the substitution (Fig. 1B,C). Consequently, loss of exon 2 resulting in loss of the canonical start site would be anticipated to significantly disrupt protein function.

The presence of homozygous *CDK4* variants was confirmed by Sanger sequencing of blood genomic DNA in all affected individuals. Parents were heterozygous carriers (Fig. 1A and Supplemental Fig. S1B), hence variant segregation in the families was in keeping with autosomal recessive inheritance. Consistent with a rare autosomal recessive disorder, both variants were observed at very low frequency in gnomAD (v4.1.0). Allele frequencies were 6.2 × 10^-7^ and 1.9 × 10^-6^ respectively, and only observed in the heterozygous state. Constraint data from gnomAD indicate *CDK4* is intolerant to biallelic loss of function variants (pNull 5×10^-4^), supporting the pathogenicity of these variants.

Clinically, all individuals presented with extreme microcephaly (occipital-frontal circumference, OFC –5.6 ± 1 SD) and most had significant postnatal growth restriction (height –2.22 ± 0.97 SD) (Fig. 1D, Tables 1,2). At birth, growth parameters were mildly reduced (OFC –1.23 ± 0.65 SD; Birth Length –0.7 ± 1.1 SD), but within normal population limits. Neuroimaging studies demonstrated significant reduction of brain size and simplified cortical gyration evident on coronal and axial projections (Fig. 1E and Supplemental Fig. S1C), in keeping with the microcephaly with simplified gyri seen in primary microcephaly (Verloes et al. 1993; Passemard et al. 2009). Cortical structural abnormalities were not evident. A hypoplastic pons without reduction in cerebellar size was reported in A.III.7 (Supplemental Fig. S1C). However, this was a variable feature, as microcephaly with simplified gyri and no brainstem alteration was reported in individual A.III.5.

Mild to moderate intellectual disability was manifest in the four affected individuals from Family A, while individual B.II.1, with the mildest microcephaly (-3.7 SD), had normal cognitive function. No distinctive dysmorphism or malformations were evident across the two families. Siblings A.III.2 and A.III.3 had 3 and 2 cafe au lait patches, respectively. They also had low insertion of the columella but were facially otherwise unremarkable (Fig. 1F). A.III.2 and an unaffected sibling had autoimmune hypothyroidism. A.III.3 had autoimmune-mediated neutropenia. Siblings A.III.5 and A.III.7 both presented delayed bone age, while A.III.7 had coeliac disease and autoimmune hypothyroidism (Tables 1,2). Individual B.II.1 developed epilepsy during childhood and exhibited mild microcytic/normocytic anaemia of undefined etiology that resolved spontaneously post-puberty (Supplemental Table S1). During a long follow-up period, slightly elevated TSH values with normal thyroid hormone, ultrasound and negative antibodies were observed (Table 2). He had a shawl scrotum, normal-sized penis and normally positioned urethra. The patient entered puberty at the age of 11, with elevated gonadotropin and normal testosterone levels. In adulthood, he has reduced testicular volume (11 mL), normal sperm analysis, and has recently fathered a healthy child.

Altogether, we identified homozygous variants in two phenotypically similar families with affected individuals displaying nonsyndromic microcephaly and short stature, which appeared likely to result in abrogation of CDK4 function. We therefore proceeded to determine the functional impact of the *CDK4* variants at the cellular level.

### Transcriptional consequences of *CDK4* mutations

**Figure 2:**
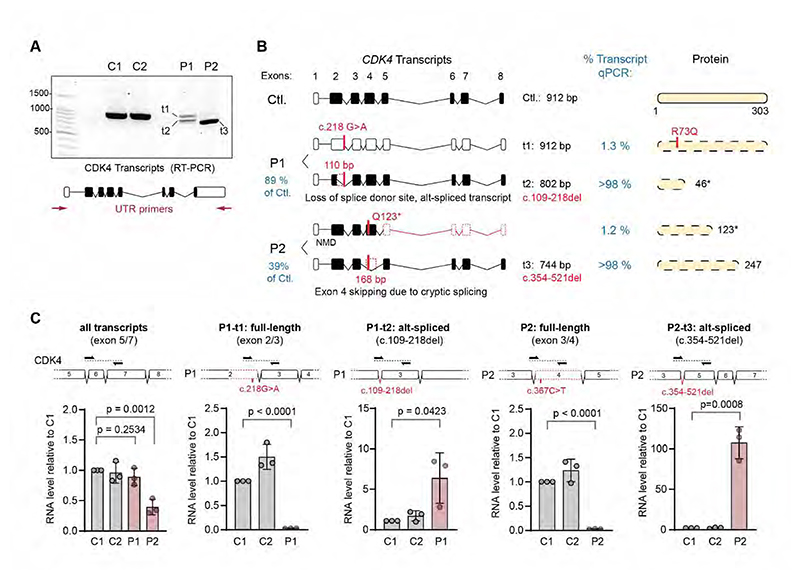
Transcriptional consequence of *CDK4* mutations. (*A*) Transcript analysis by RT-PCR of RNA extracted from primary fibroblasts. Agarose gel electrophoresis of RT-PCR products using *CDK4* 5’ UTR and 3’ UTR primers. A full-length (t1) transcript of 912 bp, and a shortened (t2) one were seen in P1 (v2), while P2 (v1) exhibits a predominant smaller transcript (t3). (*B*) Schematics of detected transcripts and their relative quantification (% Transcript) based on qPCR results presented in C; the corresponding predicted proteins are shown to the right. Supplemental Fig. S2 presents Sanger sequences of cloned *CDK4* transcripts after RT-PCR. (*C*) qPCR analysis of WT control (C1, C2) and patient specific *CDK4* transcripts relative to control. Primer locations for each qPCR reaction are indicated above each bar-graph; n = 3 experiments, mean ± SEM, two tailed t-tests.

To assess the consequence of these candidate variants on *CDK4* transcriptional levels and splicing, we established primary fibroblast cell lines from affected individuals P1 (B.II.1) and P2 (A.III.5). We investigated *CDK4* transcript levels and splicing by reverse transcription and PCR amplification (RT-PCR) of RNA extracted from P1 and P2 fibroblasts using 5’ and 3’ UTR primers. This demonstrated the presence of shorter *CDK4* transcripts that were not present in wild-type controls (Fig. 2A). Subsequent cloning and Sanger sequencing of these PCR products demonstrated two transcripts (t1, t2) in P1, one full-length *CDK4* (912 bp) containing the c.218 G>A point substitution, and a smaller 802 bp fragment with a 110 bp deletion comprising bases 109 to 218, corresponding to most of exon 2 (Fig. 2A,B and Supplemental Fig. S2A,B). The donor splice site located 1bp downstream of c.218 was disrupted as predicted by *in silico* analysis (Splice AI, Δ = 0.79) (Fig. 1C). The resulting open reading frame led to a frameshift encoding a truncated 46 a.a. length polypeptide (r.109_218del; p.Val36Alafs10), missing almost the full kinase domain (Fig. 2B and Supplemental Information). Further qPCR analysis supported the truncated polypeptide being the major transcript in this patient (Fig. 2C). In contrast, full length transcripts containing the c.218 G>A substitution (encoding the p.R73Q change) were present at negligible levels (1.3% of wild-type, Fig. 2C, second panel). Nevertheless, if it were to contribute to CDK4 protein production, the R73 sidechain participation in salt bridges would be disrupted by this mutation, suggesting that any protein derived from this R73Q allele would be functionally compromised (Supplemental Fig. S1D).

For P2, the c.367C>T substitution encoding stop codon p.Q123* was expected to cause nonsense mediated decay, and consistent with this, full length transcript (912bp) was nearly undetectable by RT-PCR (Fig. 2A). However surprisingly, a shorter transcript was present at ∼31% of wild-type transcript levels when quantified by qPCR (Fig. 2C, first graph). Sequencing of this 744 bp PCR product demonstrated a 168 bp deletion from c.354 to c.521, corresponding to Exon 4 (Fig. 2B Supplemental Fig. S2C). Thus, the predominant *CDK4* transcript in P2 cells results in an in-frame deletion with loss of 56 a.a. (p. Asp119_Val174del) (Fig. 2C, last two panels). Mapping of these 56 a.a. on the CDK4 structure shows that a key region of CDK4, including the essential activation segment (containing Thr172, critical for activity) is missing, and likely leading also to loss of protein stability (Supplemental Fig. S1E).

### CDK4 kinase is undetectable in patient-derived cells

**Figure 3:**
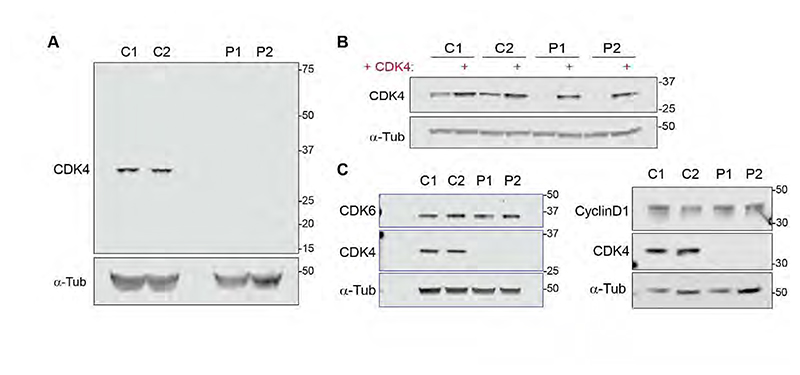
Full length CDK4 protein is undetectable in patient fibroblasts. (*A*,*B*) Immunoblots of total cell extracts obtained from exponentially growing control (C1, C2) and patient (P1, P2) fibroblasts without (*A*) and with (*B*) CDK4 complementation; loading control, alpha-Tubulin (*A*,*B*). A rabbit monoclonal antibody to C-terminal CDK4 was used; a different mouse CDK4 antibody raised against full-length CDK4 is used in Fig. 5A. A smaller ∼12KD molecular weight band is variably detected in P1 with this antibody (Supplemental Fig. S2D) that might correspond to the 46 a.a. truncated non-functional protein predicted from RNA studies. (*C*) CDK6 and Cyclin D1 levels are unchanged in patient fibroblasts compared to wild-type controls.

We next assessed the expected consequences on cellular protein levels experimentally. Here, Western Blot (WB) analysis of total cell extracts showed undetectable levels of full-length CDK4 protein in patient-derived cells from both families in contrast to control fibroblasts (Fig. 3A). CDK4 complementation confirmed specificity of CDK4 antibodies (Fig. 3B). Using another antibody raised against full-length CDK4, a smaller protein migrating between 8 and 15 kDa was detected in P1 fibroblasts, on some blots but not others. This might conceivably correspond to the 46 a.a. polypeptide (expected molecular weight 5 kDa) predicted for P1-t2, (Supplemental Fig. S2D).

In conclusion, for P1 RNA and protein analysis confirmed near complete disruption of the exon 2 splice donor site and demonstrated that the major remaining transcript had a much shorter open reading frame that led to early truncation of the CDK4 protein. Therefore, this mutation resulted in no detectable full-length CDK4 protein by immunoblotting, although expression of a small truncated CDK4 protein fragment without expected functionality, cannot be completely excluded. For P2, the full-length transcript containing the premature stop codon was markedly reduced, and the alternate transcript caused an in-frame deletion. Neither transcript produced an active kinase and therefore for P2, this mutation as for P1 should be functionally null, at the very least in terms of its canonical activity as a kinase.

### Normal mitosis in CDK4 mutant cells

A homozygous *CDK6* missense mutation (p.Ala197Thr) was previously reported in a family with primary microcephaly. While this variant did not affect CDK6 stability, centrosomal localisation of CDK6 observed in control fibroblasts was reported as lost in *CDK6* p.Ala197Thr patient cells. Additionally, patient cells displayed disorganised mitotic spindles and microtubules, supernumerary centrosomes, and nuclei with abnormal morphology, leading the authors to propose that centrosome and microtubule dysfunction contributed to the proliferation defect of *CDK6* mutant cells (Hussain et al. 2013).

**Figure 4:**
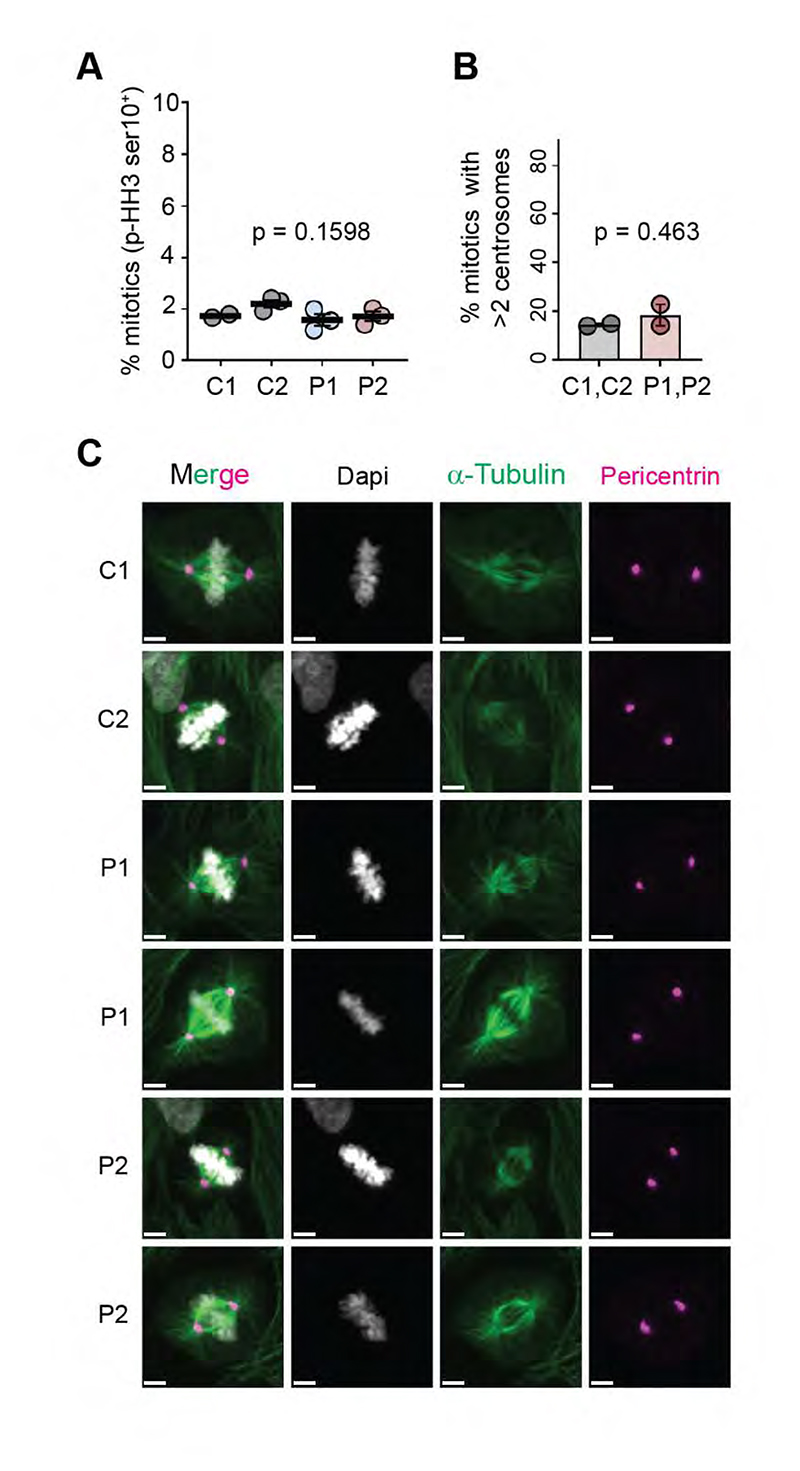
*CDK4* mutations do not alter mitosis. (*A*) Percentage of mitotic cells (p-Histone H3 ser10 positive) in control (C1, C2) and patient (P1, P2) fibroblasts measured by flow cytometry. Data points from 3 independent experiments (2 for C1); One-way Anova with Tukey post-test, mean ± SEM. (*B*) Quantification of metaphase cells with more than 2 (>2) centrosomes, expressed as percentage; Numbers of cells analysed: C1, 79; C2, 94; P1, 150; P2, 101. Two tailed t-test, mean ± SEM; measurements pooled from 2 independent experiments. (*C*) Representative confocal images of control (C1, C2) and patient (P1, P2) fibroblasts fixed and stained for Dapi (grey), α-tubulin (green) and pericentrin (magenta); scale bar, 5 μm.

Given the functional overlap of CDK6 and CDK4, we investigated the possibility of centrosome/mitotic defects in *CDK4* mutant cells. However, the proportion of mitotic cells (p-Histone H3 positive) was similar between control and patient-derived fibroblasts, suggesting that mitosis progression is unaffected (Fig. 4A). Additionally, in contrast to CDK6, CDK4 did not associate preferentially with centrosomes in human primary fibroblasts (Supplemental Fig. S3). Furthermore, mitotic spindles appeared normal as assessed by alpha-tubulin and pericentrin immunofluorescence, and we failed to detect supernumerary centrosomes in patient cells (Fig. 4B,C). Therefore, mitotic defects were unlikely to be the cause of the growth defect caused by *CDK4* microcephaly mutations.

### *CDK4* mutations impair cell proliferation and the G1/S transition

**Figure 5:**
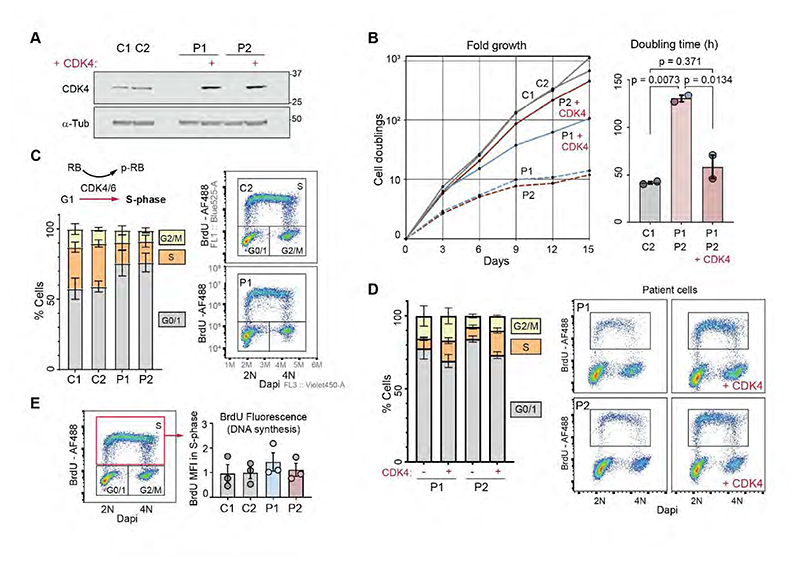
*CDK4* mutations impair G1 to S progression and lead to reduced cell proliferation. (*A*) Western blot of control and patient derived fibroblasts with/without WT CDK4 complementation. (*B*) Growth curves of control and patient derived fibroblasts w/out WT CDK4 complementation. Bar graph (right), quantification of doubling times; One-way Anova with Tukey post-test p values indicated; mean ± SEM. (*C*) Cell cycle distribution (G0/1, S, G2/M) derived from BrdU and DNA (Dapi) flow cytometry scatter plots show fewer cells in S-phase (BrdU^+^) in patient-derived fibroblasts compared to controls; n = 3 independent experiments, mean ± SEM; gates are shown on representative plots on the right. (*D*) Cell cycle distribution after complementation of patient-derived fibroblasts with CDK4. Reduced G0/G1 and increased S-phase populations consistent with rescue of a G1/S progression defect. n = 3 independent experiments, mean ± SEM; see also Supplemental Fig. S4A). (*E*) Quantification of DNA synthesis rate (BrdU Mean Fluorescence Intensity [MFI] of gated population in red rectangle) from experiments depicted in panel C.

Given CDK4’s canonical role in cell cycle progression (Baker et al. 2022; Fassl et al. 2022), we next investigated whether the *CDK4* mutations lead to reduced cell proliferation. Growth rates were determined for patient-derived fibroblasts with and without CDK4 complementation (Fig. 5A,B). CDK4 deficient cells proliferated 3 times slower than control fibroblast cell lines (doubling times: C1 = 43h, C2 = 40h, P1 = 127h and P2 = 133h). Importantly, complementation of patient cells significantly rescued proliferation, establishing CDK4 deficiency as the cause of the cell proliferation defect (Fig. 5A,B).

Since the outcome of CDK4/6 kinase function is G1 to S-phase transition (Chung et al. 2019; Yang et al. 2020), *CDK4* mutations would most likely result in accumulation of cells in G1 (Tsutsui et al. 1999), consistent with a longer cell cycle in CDK4 deficient cells. Indeed, flow cytometry experiments using a 40 min BrdU pulse to label replicating cells demonstrated that CDK4 deficient fibroblasts accumulate in G0/G1 and have proportionately fewer S-phase cells compared to control lines (Fig. 5C). Moreover, CDK4 complementation attenuated this G1 accumulation and enhanced S-phase cell numbers (Fig. 5D, Supplemental Fig. S4A). S-phase itself did not appear to be impacted, with normal levels of DNA synthesis measured by BrdU incorporation with equal Mean Fluorescence Intensity (MFI) in S-phase for wild-type and CDK4-deficient cells (Fig. 5E). Moreover, serial labelling of cells with BrdU and EdU did not detect a difference in S-phase length (Supplemental Fig. S4B). Therefore, loss of CDK4 leads to an extended G1-phase in patient cells, without detectable impact on S-phase and DNA replication. CDK6 also promotes G1/S transition, its overexpression compensating for CDK4 loss (Supplemental Fig. S5A-C). However, CDK6 and Cyclin D1 levels were unchanged in CDK4-mutant cells (Fig. 3C) and therefore such compensation did not occur in patient cells. Nevertheless, CDK6 activity within these cells was necessary for CDK4-deficient cells to progress from G1 to S, as treatment with the CDK4/6 inhibitor (CDK4/6i) Palbociclib, or CDK6 depletion by RNAi, prevented DNA replication, as measured by EdU incorporation (Supplemental Fig. S5D-F).

**Figure 6:**
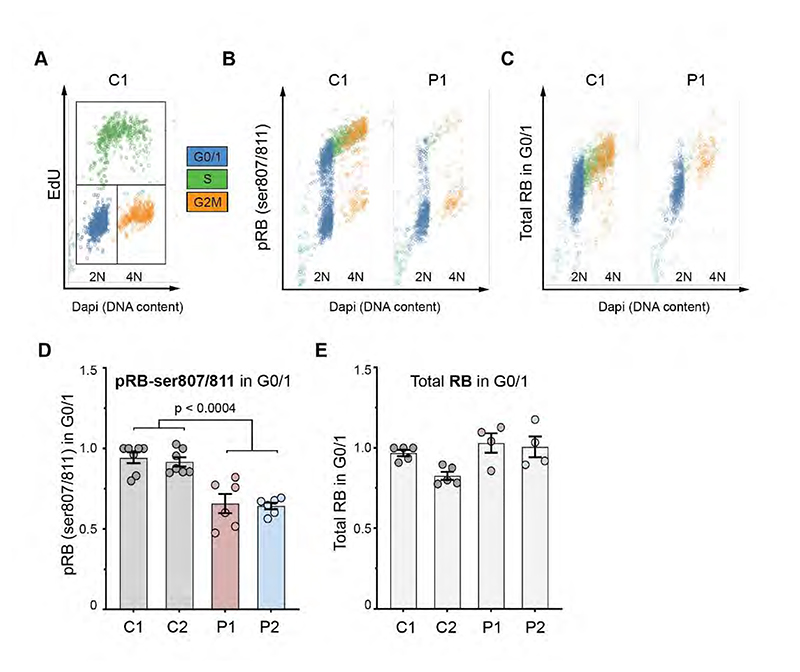
*CDK4* mutations impair Retinoblastoma phosphorylation in G1. (A-E) Quantitative image based cytometry (QIBC), (*A*) Gating strategy for cell cycle stages by DNA content (Dapi) and EdU incorporation. (*B*) Representative Dapi versus pRB-ser807/811 scatter plots demonstrate impaired RB phosphorylation in G1 in CDK4-deficient cells (P1) relative to Control 1. (*C*) Representative scatter plot of total RB levels, demonstrates equivalent levels of RB between C1 and P1. Data points for C,D individual cells, n > 1500 cells/sample in each independent experiment. (*D, E*) Quantification of pRB-ser807/811 (A) and total RB (B) fluorescence intensity per nucleus show significantly reduced pRB-ser807/811 and normal total RB levels in G0/1 in CDK4-deficient fibroblasts relative to controls; mean ± SEM (n≥4 independent experiments with 72 images/condition totalling ≥1500 cells/sample or condition in each experiment analysed).

G1 transition to S-phase is regulated by Retinoblastoma (RB) phosphorylation by CDKs (Harbour et al. 1999; Narasimha et al. 2014), which leads to de-repression of E2F transcription factors and expression of S-phase proteins such as Cyclin E and CDC25A (Leone et al. 1998; Bracken et al. 2004). Consistent with CDK4 loss impacting E2F-dependent transcription, qRT-PCR demonstrated reduced *CDC6* and *PCNA* expression (Supplemental Fig. S4C). Likewise, complementation of CDK4-deficient fibroblasts with wild-type CDK4 significantly increased expression of these E2F target genes (Supplemental Fig S4D). We next assessed RB phosphorylation, where to ensure that cell cycle was not a confounder, quantitative image-based cytometry (QIBC) (Toledo et al. 2013) was performed using a validated immunofluorescence methodology to assess pRB and RB levels in individual cells (Chung et al. 2019). Using this approach, analysis of G0/1-phase cells demonstrated a significant reduction of pRB but not RB in CDK4 deficient cells before S-phase onset, when RB phosphorylation is CDK4/6-dependent (Fig. 6A-E). pRB levels in G0/1 were rescued by CDK4 complementation and were sensitive to CDK4/6 inhibition (Supplemental Fig. S6A-D), supporting this conclusion. In S-phase, pRB levels in individual cells were similar to wild-type cells (Supplemental Fig. S6E), likely due to the subsequent feedforward activation of CDK2-CyclinA/E augmenting RB phosphorylation (Knudsen and Knudsen 2008). This could account for normal levels of DNA synthesis and S-phase duration in CDK4-deficient cells (Fig. 5E, Supplemental Fig. S4B). Together, these results indicate that the canonical function of CDK4 regulating G1 to S transition, through phosphorylation of RB in G1, underlies the cell proliferation defect caused by *CDK4* mutations.

## Discussion

CDK4 and CDK6 are functionally redundant kinases promoting cell cycle progression and the G1/S transition, linking extracellular growth signals with cell proliferation (Malumbres and Barbacid 2009; Fassl et al. 2022). Many cancer types are dependent on these activities for growth (Gao et al. 2020), and small-molecule CDK4/6 inhibitors are widely studied in clinical trials, or have shown success in the treatment of some cancer types (Fassl et al. 2022; Morrison et al. 2024).

Previously, heterozygous germ-line mutations in *CDK4* (R24C or R24H) causing gain-of-function were described in familial melanoma cases (Zuo et al. 1996; Puntervoll et al. 2013). These reduce CDK4 binding to p16^INK4^, leading to constitutive CDK4 kinase activity (Wolfel et al. 1995). Here we present evidence for CDK4 as a regulator of human brain and organism growth, identifying homozygous loss of function *CDK4* mutations in individuals from two independent families with postnatal growth restriction and severe microcephaly. *CDK4* mutations resulted in no detectable functional protein in patient fibroblasts, impairing RB phosphorylation and G1/S transition. These findings indicate that these mutations are loss of function, and that canonical CDK4 kinase dysfunction is likely responsible for the cell proliferation defect causing growth deficiency in affected individuals. Therefore, human CDK4 is not redundant with CDK6 for growth during development.

The extreme microcephaly in *CDK4* individuals, as well as the mild intellectual disability, are characteristic features of primary microcephaly (Farcy et al. 2023). However, growth parameters at birth, including OFC, were only 1-2 SD below population mean. Notably, heterozygous frameshift and nonsense variants in *CCND2* (encoding Cyclin D2), the partner of CDK4/6 with major roles in neural progenitor proliferation (Glickstein et al. 2009), also result in microcephaly (Pirozzi et al. 2021). *CDK4* and most *CCND2* microcephaly individuals display similar growth restriction, with reduced growth parameters at birth of -1 to -2 SD but more significant microcephaly postnatally (Pirozzi et al. 2021), suggestive of a prenatal-onset origin with a delayed presentation. This trajectory of early postnatal brain growth restriction has also been reported in individuals affected by paradigmatic primary microcephaly genes such as *ASPM* (Passemard et al. 2009). Therefore, while strictly not fulfilling the criteria for ‘primary’, where microcephaly is evident at birth, the *CDK4* phenotype otherwise parallels primary microcephaly and overlaps with microcephalic dwarfism.

*Cdk4* knock-out mice display significantly reduced body size, impaired fertility and insulin-deficient diabetes due to abnormal postnatal islet cell development (Rane et al. 1999; Tsutsui et al. 1999; Moons et al. 2002; Martin et al. 2003; Mettus and Rane 2003). However, B.II.1 is fertile and, while he had borderline glycosylated haemoglobin levels, none of the individuals assessed had a diagnosis of diabetes mellitus. In family A, several members had autoimmune disorders (hypothyroidism or neutropenia). However, these conditions did not entirely co-segregate with affected status and were not reported in Family B. So, they are most likely coincidental, reflecting familial aggregation for an independently acting polygenic predisposition. Cell size was not reduced in organs from *Cdk4* KO mice, indicating a primary cell proliferation defect as the source of dwarfism (Martin et al. 2003). In contrast, *Cdk4*-R24C gain-of-function (GOF) mice display increased proliferation, body size and cancer risk (Rane et al. 2002), supporting the notion that CDK4 is a bona fide regulator of cell number in mammals. While present at birth, the growth defect in *Cdk4* KO mice becomes more severe during postnatal development (Rane et al. 1999; Tsutsui et al. 1999). Hence, progressive growth restriction during development is a consistent phenotype between mice and humans.

With CDK6 primary microcephaly previously reported (Hussain et al. 2013), discovering CDK4 mutations as a cause for microcephaly seems on the face of it unsurprising. However, despite substantial overlap in function during G1-phase progression there are significant differences in disease mechanisms for growth and microcephaly associated with mutations in these two kinases. Here, we demonstrate human *CDK4* primary cells to have absent functional protein, impaired G1/S progression and cell proliferation not compensated by endogenous CDK6 activity. Hence, resulting hypocellularity likely accounts for microcephaly and growth failure in *CDK4* cases (Klingseisen and Jackson 2011). Notably, such short stature does not occur in *CDK6* primary microcephaly (Hussain et al. 2013). Likewise in mice, rather than a 50% reduction in *Cdk4* adult size (Rane et al. 1999), body weight is only slightly reduced in *Cdk6* females (Malumbres et al. 2004). Also, the homozygous Ala197Thr missense mutation does not affect CDK6 protein stability and an alternative pathogenic role for CDK6 in centrosome and microtubule organisation was proposed as causal for microcephaly (Hussain et al. 2013). While Cdk6 localisation to centrosomes was not detected in radial glia, the Ala197Thr mutation was found to impair outer Radial Glia (oRG) progenitor expansion (Wang et al. 2022). Such expansion was driven by Cdk6 in a non-catalytic manner, and was not impacted by Cdk4 deletion (Wang et al. 2022). Therefore, extreme microcephaly observed in both *CDK6* and *CDK4* cases do not share a common mechanism.

Experimental manipulation of G1 length, through Cdk4/CyclinD1 overexpression or shRNA depletion, alters the balance of progenitor self-renewal and neurogenesis, altering surface area of the postnatal cerebral cortex (Lange et al. 2009), and thus a role for CDK4 in regulating G1 progression in neurogenesis would be sufficient to explain microcephaly. However, because these experiments included the simultaneous overexpression/depletion of Cyclin D, they do not preclude a role for the CDK6 kinase.

Despite the functional equivalence of Cyclin D1, D2 and D3 in supporting CDK activity, D-type Cyclin knockouts have differing developmental consequences, (Ciemerych et al. 2002; Glickstein et al. 2009; Saleban et al. 2023). This may be explained, at least in part, by different tissue expression patterns. Likewise, interrogation of a published scRNAseq dataset (Telley et al. 2019) demonstrates distinct expression patterns for CDK4 and CDK6 during neurogenesis (Supplemental Fig. S7). Notably, CDK6 expression is substantially more restricted to early progenitors, with CDK4 expression persisting longer during neurogenic differentiation. This raises the possibility that loss of CDK4 may become limiting later in neurogenesis when CDK6 expression is minimal or absent. Also, the predominantly postnatal nature of *CDK4* microcephaly suggests that other processes contributing to brain volume could be impacted, such as gliogenesis, which peaks in the 3^rd^ trimester and continues postnatally (Spalding et al. 2005; Gilmore and Walsh 2013). Furthermore, CDK4 might have a non-canonical role, analogous to the role of CDK6 in cilia regulation (He et al. 2025) or the unconventional role of cyclin-dependent kinase CDK5 in post mitotic neurons (Tsai et al. 1993).

With LOF mutations in the CDK4/6 binding partner *CCND2* causing microcephaly (Pirozzi et al. 2021), and GOF *CCND2* mutations leading to brain overgrowth (Mirzaa et al. 2014), we conclude that CDK4 and G1 Cyclin-CDK activity represent a key axis controlling human brain growth. A limitation of our study is that CDK4 loss has not been investigated in neural progenitors, hence its neurodevelopmental mechanism remains to be confirmed, and the cell populations impacted to be defined. Cortical size is much more subtly affected in microcephaly mouse models (Pulvers et al. 2010) and has not been investigated in *Cdk4* KO mice. Given our findings in humans here, re-evaluation of *in vivo* neurogenesis in *Cdk4*^*-/-*^ mice is warranted, as a role for CDK4 in corticogenesis may have been previously overlooked.

## Materials and Methods

### Research subjects

Patients were recruited to research studies at the MRC Human Genetics Unit, University of Edinburgh, UK and the University of Sao Paulo, Brazil by their local clinician. The research studies were approved by the Multicentre Research Ethics Committee for Scotland (05/MRE00/74), and Ethics Committee of Hospital das Clinicas da Faculdade de Mediciana da Universidade de Sao Paulo (37868114.3.0000.0068), respectively. Informed written consent was obtained from all participating families. Families provided written consent for the publication of clinical photographs.

### DNA sequencing and variant validation

Genomic DNA was extracted from peripheral blood by standard methods. Trio whole genome sequencing for A.III.2 and A.III.7 was performed at Edinburgh Genomics utilising Illumina SeqLab, which integrates Illumina TruSeq library preparation, Illumina cBot2 cluster generation, Illumina HiSeqX sequencing, Hamilton Microlab STAR integrative automation, and Genologics Clarity LIMS X Edition. Detailed methods for DNA QC, library preparation and sequencing can be found in the Supplemental Information file.

*Bioinformatics analysis:* Demultiplexing was performed using bcl2fastq (2.17.1.14), allowing 1 mismatch when assigning reads to barcodes. Adapters were trimmed during the demultiplexing process. BCBio-Nextgen (0.9.7) was used to perform alignment, BAM file preparation and variant detection. BCBio used bwa mem (0.7.13) to align the raw reads to the Human genome (GRCh38 with alt, decoy and HLA sequences), then samblaster (0.1.22) to mark the duplicated fragments (Faust and Hall 2014), and the Genome Analysis ToolKit (3.4-0-g7e26428) for indel realignment and base recalibration (McKenna et al. 2010). Genotype likelihoods for each sample were calculated using the GATK HaplotypeCaller and resulting GVCF files called jointly using GATK’s GenotypeGVCFs function. Variant quality score recalibration (VQSR) was performed as per GATK best practices (Van der Auwera et al. 2013), with a truth sensitivity threshold of 99.9%. Following variant calling, variant calls were annotated with Ensembl’s Variant Effect Predictor (McLaren et al. 2016) and filtered to identify rare (AF < 0.5%) functional (non-synonymous, splice site, coding indels) variants consistent with biallelic inheritance in both sequenced individuals.

Whole exome sequencing was performed on B.II.1 according to previously published protocols (Homma et al. 2019). Briefly, the library was constructed with SureSelect Human All Exon V7 Kit (Agilent Technologies, Santa Clara, USA) according to the manufacturer’s instructions. The exome library was sequenced on NovaSeq platform (Illumina, San Diego, USA) running on paired-end mode. Reads were aligned to the GRCh37/hg19 assembly of the human genome. Variant calling was performed with Freebayes (Garrison and Marth 2012) and the resulting VCF was analysed through the Franklin Genoox platform. Based on the family pedigrees indicating consanguinity, the exome data were screened for homozygous variants in index patient, in addition to being absent in gnomAD (Chen et al. 2024), ABraoM (Naslavsky et al. 2017) and SELAdb (Lerario et al. 2020) public databases, these last two being representative of the Brazilian population. Synonymous mutations were excluded. Data screening for deleterious variants were performed as previously reported (Homma et al. 2019). Candidate variants were submitted to the GeneMatcher platform (Sobreira et al. 2015) in search of additional cases with concordant genotype and phenotype. Variant interpretation followed the American College of Medical Genetics and Association for Molecular Pathology (ACMG-AMP) variant pathogenicity guideline (Richards et al. 2015).

Assessment of gene function was performed using the Online Mendelian Inheritance in Man (OMIM) and the PubMed databases.

### Sanger sequencing

Variants were confirmed by bidirectional capillary dye-terminator sequencing and annotated using the reference sequence, GenBank: NM_000075.4. Capillary sequencing was performed at the MRC Human Genetics Unit, Edinburgh, UK, and the University of Sao Paulo, Brazil. Primer sequences and PCR conditions for targeted *CDK4* sequencing are available on request.

### Splice site analysis

Variant determination was carried out using Alamut Visual Plus v.1.8 (©SOPHiA GENETICS), which uses five distinct splice site prediction algorithms: SpliceSiteFinder-like, MaxEntScan, NNSPLICE, GeneSplicer, and Human Splicing Finder. Splice AI predictions were generated using the web application (Jaganathan et al. 2019).

### Cells and cell culture

Primary dermal fibroblasts were established from skin punch biopsies and maintained in AmnioMAX medium (Thermo Fisher Scientific 17001074) in 5% CO2 and 3% O2.

### Transcript analysis by RT-PCR

Cell pellets were harvested from control and patient-derived fibroblast cell lines; RNA was extracted using the *RNeasy kit* (Qiagen Cat. No. 74004) and cDNA was generated using *SuperScript™ III First-Strand Synthesis System* (Invitrogen, Cat. No. 18080051). PCR amplified products using 5’ and 3’ UTR primers and *Phusion Flash High-Fidelity PCR Master Mix* (Thermo Scientific Cat. No. F548S) were isolated from 1% agarose gels and DNA extracted using Gel *QIAquick*®* Gel Extraction Kit* (Qiagen Cat. No. 28704). Products were cloned into a Topo vector using *Zero Blunt*™* TOPO*™* PCR Cloning Kit* (Invitrogen Cat. No. 450245). Colony DNA was obtained using *QIAprep*®* Spin Miniprep Kit* (27104, Qiagen), and colony PCRs were carried out using *DreamTaq Green PCR Master Mix* (Thermo Scientific Cat. No. K1081) and M13 Fwd and Rev primers for sequencing.

### Primers for RT-PCR

**Table:** 

	Length	Tm	GC%
Forward: GGTCTCCCTTGATCTGAGA**ATG**	22	62	50	
Reverse: TCAGTGTCCAGAAGGGAAATG	21	62	47.6	

(Transcription start site indicated in bold; the 3’ end of the Reverse primer is located 37 bp downstream of the CDK4 stop codon; ENST00000257904.11)

### RT-qPCR analysis of CDK4 splice variants and E2F target gene expression

Cell pellets were harvested from two control and patient-derived fibroblast cell lines at three independent times. RNA was extracted using *RNeasy Plus Mini Kit* (Qiagen Cat. No. 74134). cDNA was generated using *SuperScript™ III First-Strand Synthesis System* (Invitrogen, Cat. No. 18080051), using random hexamer primers. CDK4 variant expression was assessed using *SYBR Select Master Mix* (Applied Biosystems Cat. No. 4472908), according to the manufacturer’s recommendations (with T_a_ = 58°C). Primers spanning exon 5-7 were designed to amplify all potential CDK4 variants (CDK4-ctrl-FW: CGAAAGCCTCTCTTCTGTGGAAAC, CDK4-ctrl-RV: CAGGGATACATCTCGAGGCCAG). Primers spanning the exon 2/3 boundary were designed to monitor the presence of full-length CDK4 transcript in P1 (CDK4-exon2/3-FW: ACTGAGGCGACTGGAGGC, CDK4-exon2/3-RV: GGTGCCTTGTCCAGATATGTCC). To monitor the truncated transcript in P1, primers were designed targeting the c.109-218del region (CDK4-c.109-218del-FW: CCTCAAGAGTGCTGATGGACG, CDK4-c.109-218del-RV: GGTGCCTTGTCCAGATATGTCC). Primers spanning the exon 3/4 boundary were designed to monitor the presence of full-length CDK4 transcript in P2 (CDK4-exon3/4-FW: CCGAAACGATCAAGGATCTGATGC, CDK4-exon3/4-RV: CCAAAGTCAGCCAGCTTGACTG). To monitor the truncated transcript in P2, primers were designed targeting the c.354-521del region (CDK4-c.354-521del-FW: AGCCGAAACGATCAAGGTTGTTAC, CDK4-c.354-521del-RV: TTCGACGAAACATCTCTGCAAAGATAC). CDK4 complementation was measured using primers targeting a codon-optimized CDK4 sequence (CDK4-codonopt-FW#1: CCGCACGGATCGAGAAATTAAAG, CDK4-codonopt-RV#1: GGAGAAAGTCCAGACCTCGTAAG, CDK4-codonopt-FW#2: GGCAATAGTGAGGCGGATCAAC, CDK4-codonopt-RV#2: CCATTTCGGGCACTACAGATTGTAC). E2F target gene expression was monitored using primers targeting to CDC6 (CDC6-FW#1: CCACTGTCTGAATGTAAATCACCTTC, CDC6-RV#1: AAGAGGGAAGGAATCTTGTGCTC, CDC6-FW#2: CTCTGGGGAAGTTATATGAAGCCTAC, CDC6-RV#2: TCCAAGAGCCCTGAAAGTGAC) and PCNA (PCNA-FW: GCGTGAACCTCACCAGTATGT, PCNA-RV: TCCTGGTTTGGTGCTTCAAATACTAG). All reactions were normalized to GAPDH housekeeping gene (GAPDH-FW: CGGATTTGGTCGTATTGGG, GAPDH-RV: TGGGTGGAATCATATTGGAAC).

### CDK4 and CDK6 complementation

Patient fibroblasts were transduced with lentiviral particles containing pLIX_403-CDK4 and/or pLIX_403-CDK6, a construct where full-length codon-optimized CDK4 or CDK6 was gateway cloned into pLIX_403 (Addgene #41395, #158560). The human CDK4 or CDK6 clones were obtained as pENTRY vectors from Twist Bioscience. CDK4 containing cells were selected with 0.5 μg/ml Puromycin (Gibco Cat. No. A11138-03), and CDK6 containing cells were selected with 3.3 μg/ml Blasticidin (Invivogen, Cat. no. ant-bl-1).

### CDK6 RNAi

Dharmacon siGENOME smartpool against Ctl (non-targeting sequences) or human CDK6 (Cat# M-003240-02-0005) was used according to manufacturer’s instructions. Cells were transfected in 10cm or 60cm plates for 48h before trypsinization and subsequently seeded for 24h for quantitative microscopy as explained below.

### Protein modelling

For Supplemental Fig.S1D, the AlphaFold model of human CDK4 (Uniprot P11802) was visualized with PyMOL. Crystal structure of CDK4 in complex with Cyclin D3 (PDB 3G33) (Takaki et al. 2009) was visualized using UCSF ChimeraX (Meng et al. 2023).

### Immunoblotting

Total cell extracts were prepared in urea lysis buffer containing 8 M urea, 50 mM Tris-HCl, pH 7.5, 150 mM β-mercaptoethanol, protease inhibitors and PhoSTOP (Roche Cat. No. 04693132001 and 4906837001). Lysed samples were sonicated 7 × 30 sec ON/OFF cycles using a Bioruptor (Diagenode). Protein electrophoresis was performed using NuPAGE 10% or 4-12% Bis-Tris mini protein gels (Invitrogen Cat. No. NP0336BOX, NP0301BOX) and MOPS running buffer (Cat. No. NP0001) at 80-130 V. Wet transfer of proteins to Immobilon-FL PVDF membrane (Millipore Cat. No. IPFL00010) was performed at 100 V for 60-75 min at 4°C. After transfer, membranes were washed in methanol, air-dried and re-activated in methanol, washed in 1X Tris-buffered saline/0.2% Tween-20 (Sigma. Cat. No. P1379) (TBS-T), and blocked in TBS-T/2.5% BSA (Roche Cat. No. 10735086001, lot 64758420) for 1 h at room-temperature (RT). Blots were incubated overnight (O/N) in TBS-T/2.5% BSA containing primary antibody. After 4 × 5 min washes in TBS-T, blots were incubated with secondary antibodies (1:20,000-30,000) for 1 h at RT, washed 4 × 5 min in TBS-T and rinsed in TBS before acquisition using a LI-COR Odyssey® CLx imager. ImageStudio software was used for quantification.

Primary antibodies:

- CDK4 (Cell Signaling Technology Cat# 12790, RRID:AB_2631166): clone D9G3E; Rabbit monoclonal to C-terminal CDK4.
- CDK4 (Proteintech Cat# 66950-1-Ig, RRID:AB_2882273): mouse monoclonal to full length CDK4 (fusion prot. Cat. No. Ag20538).
- Mouse anti-Alpha tubulin (α-Tub), clone B-5-1-2 (Sigma Cat. No. T6074, RRID:AB_477582): lot 037M4804V, 1:10,000.
- Anti-pRB ser807/811 (CST Cat. No. 9308, RRID:AB_331472): Rabbit polyclonal, 1:2,000.
- Anti Cyclin D1 [SP4] (ab16663), 1:2,500
- Anti CDK6 (Proteintech Cat. No. 14052-1-AP), 1:2,500

Secondary antibodies for immunoblotting:

- IRDye 680RD Goat anti-Rabbit IgG (H + L) Highly Cross-Adsorbed, 0.1 mg (LI-COR Biosciences Cat# 925-68071, RRID:AB_2721181).
- IRDye 800CW Goat anti-Mouse IgG (H + L) Highly Cross-Adsorbed, 0.1 mg (LI-COR Biosciences Cat# 925-32210 (also 925-32210), RRID:AB_2687825).
- IRDye 680RD Goat anti-Mouse IgG (H + L) Highly Cross-Adsorbed, 0.1 mg (LI-COR Biosciences Cat# 925-68070, RRID:AB_2651128).
- IRDye 800CW Goat anti-Rabbit IgG (H + L) Highly Cross-Adsorbed, 0.1 mg (LI-COR Biosciences Cat# 925-32211, RRID:AB_2651127).
- IRDye 680RD Donkey anti-Mouse IgG (H + L). Highly Cross-Adsorbed, 0.1 mg (LI-COR Biosciences Cat# 925-68072, RRID:AB_2814912).

### Growth curve

Human primary fibroblasts (1.5 × 10^5^ cells) were seeded on day 0 into a T25 flask in 3% O_2_, split and counted every 3 days, and 1.5 × 10^5^ cells were reseeded into a new flask. Counts were measured in duplicate using a Countess automated cell counter according to manufacturer’s instructions. Doubling times were calculated during log-phase growth (day 3-15) using the formula: *t*/log2 (*e*/b) where t = time in hours, e = final population size and b = population size at the start of log-phase growth.

### Flow cytometry

#### BrdU incorporation

Exponentially growing cells were pulsed with 64 μM BrdU for 40 min (in pre-warmed media), rinsed once with PBS, trypsinized, pelleted at 1,200g, resuspended in 75 μl PBS and fixed by adding 1 ml 100% freezer-cold ethanol with gentle vortexing, before storage at -20°C. Fixed cells were pelleted by centrifugation at 1,300g for 5 min, then washed in 1X PBS/0.1% Triton-100-X (PBS-T).

DNA denaturation was performed in 15 ml Falcon tubes with all centrifuge steps at 300g, as described previously (Darzynkiewicz and Juan 2001). Cells were centrifuged for 10 min at 300g, resuspended in 1X PBS-T and centrifuged for 6 min. The pellet was resuspended in 1X PBS-T/0.1M HCl, incubated for 2 min at RT, then centrifuged. The pellet was resuspended in 1.5ml DNA Denaturation buffer (0.15mM NaCl, 15μM Trisodium citrate dihydrate), heated at 95C for 5 min, before being chilled immediately on ice. 5ml Antibody Diluting Buffer (ADB: 1X PBS, 0.1% Triton, 1% FBS) was added and cells were centrifuged.

For staining, cells were transferred to 1.5 ml tubes with centrifuge steps at 1200g. The pellet was resuspended in 50μl ADB plus rat anti-BrdU (Abcam, Cat# ab6326, 1:600), incubated at RT for 60 min, washed in 1ml ADB and centrifuged for 5 min. Cells were incubated in ADB plus goat anti-rat-AF488 secondary antibody (Invitrogen, Cat# A11006, 1:1500) for 45 min at 4C, washed in 1ml ABD and centrifuged for 5 min. The cells were incubated in 1ml ABD containing Dapi (1:1000; final concentration of 20 μg/ml) for 5 min at RT, then centrifuged and resuspended in 350 μl PBS.

A Cytoflex S analyzer (Beckman Coulter) was used with the Violet 405 nm laser and 450/45 bandpass filters for Dapi detection, and the 450 nm laser and 525/50 filter for BrdU detection. 10-20,000 events in the single-cell population gate were recorded. Data analysis was performed using FlowJo v10.8.1 (FlowJo LLC, BD).

#### p-Histone H3 ser10

Pellets of exponentially growing cells were suspended in 500μl 2% paraformaldehyde (PFA) in PBS and fixed 15 min on ice. 500μl PBS/0.1% Triton was added and cells spun down at 1000g, resuspended in 500μl FACS Storage buffer (3% hi FBS/PBS/0.09%Na-Azide) and at 4C for several days. Cells were stained with mouse anti-p-Histone H3 ser10 (Cat. No. CST9706 (GG3), 1:300) using the same staining procedures as shown above for flow cytometry staining.

#### Immunofluorescence

Cells were seeded in glass bottom 8-well chambers (Ibidi Cat. No. 80807) and fixed for 5 mins at room temperature (RT) in Psuc (4% PFA, 2% sucrose, PBS), followed by Methanol:Acetone (1:1) for 3 min at -80°C. To block, cells were then washed 3 × 5 mins in PBS, and incubated 1hr at RT in block solution (1% BSA/PBS, or 10% goat serum + 0.1% triton/PBS). To stain, cells were incubated with primary antibodies in block solution for overnight at 4°C, rinsed 3 × 5 min with wash solution (1% BSA/PBS, or 0.5% goat serum + 0.1% triton/PBS for fibroblast cdk4 staining), followed by incubation with secondary antibody in wash solution for 1hr at RT, and 3 × 5 min washes. To stain DNA, cells were incubated with Dapi in PBS (1:2000 1mg/mL) for 5 min at RT, then washed 3 × 5 min in PBS. Cells were stored in PBS until imaging. Pericentrin/α-tubulin staining:

Primary antibodies:

- Rabbit anti-pericentrin (Abcam, Cat# ab4448); 1:400.
- Rat anti α-tubulin (Serotec, Cat# MCA77G); 1:500.

Secondary antibodies:

- 568 goat anti-rabbit (Invitrogen, Cat# A11036); 1:500.
- 488 goat anti-rat (Invitrogen, Cat# A11006); 1:500.

Cdk4/α-tubulin staining in fibroblasts:

Primary antibodies:

- Rabbit anti-Cdk4 (Cell Signaling, Cat# 12790); 1:1000.
- Rat anti α-tubulin (Serotec, Cat# MCA77G); 1:1000.

Secondary antibodies:

- 647 goat anti-rabbit (Invitrogen, Cat# A21244); 1:2000.
- 488 goat anti-rat (Invitrogen, Cat# A11006); 1:200.

#### p-RB ser807/811 and total RB staining in fibroblasts

Cells were seeded in glass bottom 8-well chambers (Ibidi Cat. No. 80807) using 7,500 cells/well and cultured for 24-72h. 0.4 μg/ml Doxycycline and/or 0.2 μM CDK4/6 inhibitor, (Palbociclib, Cat. HY-50767-5mg, Cambridge Bioscience) or DMSO and grown for 24h before a 25 min 40 μM EdU (Sigma, Cat. No. 9000584) pulse before cell fixation in 2% PFA/PBS for 20 min at 4C. Cells were washed 3 × 5 min in PBS, permeabilised in 0.3% Triton/PBS for 15 min., and incubated 1hr at RT in block solution (5% goat serum/0.1% Triton/PBS). Cells were then incubated with microscopy-validated antibodies to *p-RB ser807/811 (CST Cat. No. 8516 – (D2B12) XP) and total RB* (CST Cat. No. 9309 – (4H1)) at 1:2,000 dilution in block solution O/N at 4°C, rinsed 3 × 5 min in 0.1% Triton/PBS, followed by incubation with secondary antibodies: AF568 goat anti-rabbit (Invitrogen, Cat# A11036) and AF488 goat anti-mouse (Invitrogen, Cat# A11029), 1:3000 in block solution for 1hr at RT. After a 5 min wash, cells were incubated for 30min at RT in EdU labelling buffer, adding the following components in order (for 1ml): 100μl 20mM CuSO4, 100μl 0.5M L-ascorbic acid, 800μl 0.1%Triton/PBS, 0.2μl Alexa fluor 647 azide. 3 × 5 min washes were followed by nuclear staining using Dapi, as shown above.

#### S-phase time by microscopy

A full protocol for S-phase time measurements shown in Supplemental Fig. S4B was previously presented (Parry et al. 2020); this time, a 64 μM BrdU pulse for 2.25 h was followed by a 15-min 40 μM EdU pulse. Cells were then fixed in 4% PFA. DNA was denatured with 1X PBS/ 0.1% Triton/ 0.1M HCl for 15 min, washed, and BrdU was detected using Mouse anti-BrdU MoBu-1 Ab8039, 1:600 O/N at 4C. The following day, AF488 Goat anti-mouse secondary antibody (1:3000) was used to detect BrdU for 1h at RT. The click reaction using AF647-Azide to detect EdU was followed as shown above for QIBC studies.

### Image analysis

Mitosis detection and pericentrin spot imaging were carried out using ScanR (Olympus). Detailed methods for widefield imaging and image analysis can be found in the Supplemental Information.

### ScanR QIBC pRB and total RB

Chamber slides were automatically imaged in ScanR widefield mode using a UPLXAPO 40X 0.95 NA objective (Olympus) using a Lumencor SpectraX LED light source (Lumencor) and Semrock Briteline DAPI/FITC/Cy3/Cy5 optical filters to detect Dapi, total RB, pRB-ser807/811 and EdU. For further details on the widefield imaging acquisition, see Supplemental Information. 72 images/well averaging 1500-2000 cells/condition were acquired and analysed for each independent experiment using the ScanR Acquisition Software (Olympus).

After background correction, nuclei were segmented using the Artificial Intelligence module for object identification, and the fluorescence intensity of each channel was generated for each object. Single cells were gated by a nuclear area/circularity gate excluded doublets, and a subsequent DNA content histogram gate using Dapi selected the single-cell population. Subsequent Dapi vs. EdU plots were used to gate cell cycle stages. G0/G1; S and G2/M, as documented in Figure 6A. pRB and RB levels were quantified for gated cell cycle populations, depicted in Fig 6 and Supplemental Fig. S6. Here, the ScanR analysis software was used to generate fluorescence intensity values per object and for each individual well and gate. The total and fraction of cells in each gate was exported from the ScanR analysis. Tableau Software (license for Researchers) was used to generate plots presented in Fig. 6. https://public.tableau.com/app/discover

### Statistical analysis

Statistical testing was performed using GraphPad Prism v.10. Two-sided parametric (t-tests), one-sample t-tests, or non-parametric Mann–Whitney *U*-tests were performed for quantitative measurements as indicated in figure legends. One-way ANOVA test was performed for cell doubling time; significance (P-values) indicated on figures or legends. Number of samples and/or experimental replicates indicated on figures or legends. Additional statistical test details (t and F values and degrees of freedom) are shown in Source Data file.

## Supplementary Material

Supplementary

## Figures and Tables

**Table 1:** Biallelic *CDK4* variants in individuals with microcephaly and short stature

Family	Individual	Sex	Nucleotide change	Amino acid consequence	Allele freq. (gnomAD)	Segregation	Consanguinity	OFC (SD)	Height (SD)	Intellectual disability#	Age at Exam	Other
A	A: III.2	F	c.367C>T	p.Gln123*	6.20 × 10^-07^	M, P	Yes	-5.8	-0.5	Mild	11y 8m	Autoimmune Hypothyroidism
A	A: III.3	M	c.367C>T	p.Gln123*	6.20 × 10^-07^	M, P	Yes	-5.5	-2.3	Mild	6y 1m	Autoimmune Neutropenia
A	A: III.5 (P2)	F	c.367C>T	p.Gln123*	6.20 × 10^-07^	M, P	Yes	-6.8	-3.4	Mild	19y 6m	
A	A: III.7	F	c.367C>T	p.Gln123*	6.20 × 10^-07^	M, P	Yes	-6.2	-2.8	Mild	10y 3m	Coeliac disease, Autoimmune hypothyroidism, pons hypoplasia
B	B: II.1 (P1)	M	c.218G>A	p.R73Q and Loss of essential donor splice site	1.86 x10^-06^	M, P	Yes	-3.7	-2.1	No	33y 8m	Epilepsy, anaemia, low reticulocytes

Variants are described using HGVS nomenclature (https://hgvs-nomenclature.org/stable/recommendations/general/) for the reference coding DNA (HGVSc, NCBI reference sequence NM_00075.4), and protein (HGVSp, NCBI reference sequence NP_000066.1) sequences. Variant allele frequency (AF) for Total gnomAD v4.1 population. DNA variants are expressed relative to the coding (“c.”) sequence, and all protein changes are preceded by “p.” OFC and Height at latest evaluation shown; # additional clinical information in Table 2.

**Table 2:** Detailed clinical features of individuals with *CDK4* variants and microcephaly

Individual:	A: III.2	A: III.3	A: III.5	A: III.7	B: II.1
*CDK4* variant	c.367C>T; p.Gln123Ter	c.367C>T; p.Gln123Ter	c.367C>T; p.Gln123Ter	c.367C>T; p.Gln123Ter	c.218G>A
Ethnicity	British Pakistani	British Pakistani	British Pakistani	British Pakistani	Brazilian
Consanguinity	Yes (1^st^)	Yes (1^st^)	Yes (1^st^)	Yes (1^st^)	Yes (1^st^)
Mid parental height SDS	160 cm / -0.6 SD	173cm / -0.6 SD	155cm / -1.4 SD	155cm / -1.4 SD	180cm / 0.8 SD
Sex	Female	Male	Female	Female	Male
Current age	11y	6y	19y	10y	33y
Prenatal-onset growth restriction	No – maternal gestational diabetes	No	No	No	Yes
Gestational age (w)	40	40	38	40	39
Birth weight SDS	3.44 kg / 0.1 SD	3.08 kg / -1.1 SD	2.6kg / -1.0 SD	3.18kg / -0.6 SD	2.68 kg / -1.6 SD
Birth length SDS	53.5 cm (at 1mo) / -0.1 SD	50 cm / -0.4 SD	49.5cm / 0.4 SD	49cm / -0.6 SD	44 cm / -2.8 SD
Birth OFC SDS	33.5 cm / -0.8 SD	34 cm / -0.4 SD	32 cm / -2.0 SD	32.5cm / -1.7 SD	NA
**At the first evaluation**					
Postnatal growth retardation	Yes	Yes	Yes	Yes	Yes
Microcephaly	Yes	Yes	Yes	Yes	Yes
Chronological age (y)	1	0.75	0.83	4.58	4.1
Height SDS	67 cm / -2.8 SD	62 cm / -4.1 SD	66 cm / -2.2 SD	92.4 cm / -3.1 SD	92.5 cm / -2.5 SD
Weight or BMI SDS	7.3 kg / -1.7 SD	6.98 kg / -2.2 SD	5.66 kg / -3.4 SD	11.8 kg / -3.4 SD	13.3 kg / -2.1 SD
OFC SDS	39.9 cm / -5.6 SD	40.4 cm / -5.1 SD	37.5 cm / -7.1 SD	43.9 cm / -6.3 SD	43.5 cm / -6.0 SD
**Age at most recent exam**	11y 8m	6y 1m	19y 6m	10y 3m	32y 8m
Recent height (cm)	144.4 cm / -0.5 SD	105 cm / -2.3 SD	143.4 cm / -3.4 SD	121.5cm / -2.8 SD	163.7cm / -2.1 SD^1^
Recent weight (kg)	43.6 kg / 0.6 SD	15.85 kg / -2.4 SD	33.39 kg / -4.6 SD	17.4 kg / -4.5 SD	54.6 kg / -2.0 SD
Recent OFC (cm)	46.8 cm / -5.8 SD	44.8 cm / -5.5 SD	46.1 cm / -6.8 SD	46.0 cm / -6.2 SD	51.0 cm / -3.7 SD
Developmental delay	Learning disability diagnosed on formal cognitive assessment*	Speech delay, behind peers at school	Mild to moderate learning disability diagnosed on formal cognitive assessment	Learning disability diagnosed on formal cognitive assessment	No
Dysmorphic features / congenital malformations	Low insertion of columella, 3 café au lait patches	Low insertion of columella, 2 café aulait patches	Preauricular skin tag		Shawl scrotum, small testicles in adulthood
Other clinical features	Autoimmune Hypothyroidism	Consumptive neutropenia	Growth did not respond to growth hormone, delayed bone age (4.0) at chronological age 8.0	Coeliac disease, delayed bone age (2.8) at chronological age 4.5 and bone age 5.0 at chronological age 7.5, Autoimmune Hypothyroidism	Epilepsy during childhood; normocytic anemia with low reticulocyte^2^
Laboratory findings					
FT4 pmol/L	**8.2** (n range 12-22)	15 (n range 10-18)	10 (n range 10-19)	11.4 (n range 12-22)	14 – 20 (n range 10-19)
TSH mU/L	**5.1** (n range 0.27-4.21)	3.4 (n range 0.5-4.2)	3.41 (n range 0.5 -3.9)	8.7 (n range 0.27-4.2)	7.7 to 12.4 (n range 0.4- 4.5)
GH peak at a stimulation test μg/L	NA	NA	20	NA	8.8
IGF-1 ng/L	NA	93 (n range 28-247)	115 (n range 35-240)	51 (n range 25-198)	**46** (n range 81-280)
LH/FSH IU/L	5.7/ 5.5	NA	NA	NA	35.7 / 18.4^3^
Other labs	Anti Thyroid Peroxidase 204.0 IU/ml (n range <34.0) Urine OA, AA, MPS normal, Fanconi Breakage studies normal	Granulocyte specific antibodies; normocellular marrow with adequate myeloid precursors which are maturing to segmented neutrophils	HbA1c 37mmol/mol (n range 20-41), glucose 4.3 mmol/L (n range 3.3 – 6.1)	Anti Thyroid Peroxidase 165.0 IU/ml (n range <34.0) HbA1c 37mmol/mol (n range 20 – 41), random glucose 5.1mmol/L (n range 3.3 – 6.1)	Normal blood glucose levels with an HbA1c of **5.7-6.0%**
Brain MRI findings	NA	NA	Brain MRI normal	Microcephaly with simplified gyri. Hypoplastic pons with relatively normal cerebellum. Dorsal and ventral midline clefts of pons and medulla, small cerebellar peduncles.	Rathke cleft cyst resolved^4^

NA: data not available; 1: Adult height after 5.6 year of rhGH therapy; 2: Normal hemoglobin electrophoresis and iron status; 3: In puberty and adulthood with normal testosterone levels [18.4 to 28.8 nmol/L (normal range 10 to 35 nmol/L)] and spermogram count, normal fertility; 4: Rathke cleft cyst was observed at the age of 8 and completely disappeared during the follow-up (last image at the age of 18).

* A: III.2: psychology assessment meets criteria for learning disability.

## Data Availability

WGS data will be deposited at the European Genome Archive on publication for individuals from Family A. WES data for Family B is not consented for deposition. Source data are provided with the manuscript.
